# Neural correlates of phonetic convergence and speech imitation

**DOI:** 10.3389/fpsyg.2013.00600

**Published:** 2013-09-11

**Authors:** Maëva Garnier, Laurent Lamalle, Marc Sato

**Affiliations:** ^1^GIPSA-LAB, Département Parole et Cognition, CNRS et Grenoble Université, UMR5216Grenoble, France; ^2^Inserm US 017Grenoble, France; ^3^IRMaGe, Université Grenoble AlpesGrenoble, France; ^4^CHU de Grenoble, UMS IRMaGeGrenoble, France; ^5^CNRS, UMS 3552Grenoble, France

**Keywords:** phonetic convergence, imitation, speech production, speech perception, sensory-motor interactions, internal models

## Abstract

Speakers unconsciously tend to mimic their interlocutor's speech during communicative interaction. This study aims at examining the neural correlates of phonetic convergence and deliberate imitation, in order to explore whether imitation of phonetic features, deliberate, or unconscious, might reflect a sensory-motor recalibration process. Sixteen participants listened to vowels with pitch varying around the average pitch of their own voice, and then produced the identified vowels, while their speech was recorded and their brain activity was imaged using fMRI. Three degrees and types of imitation were compared (unconscious, deliberate, and inhibited) using a go-nogo paradigm, which enabled the comparison of brain activations during the whole imitation process, its active perception step, and its production. Speakers followed the pitch of voices they were exposed to, even unconsciously, without being instructed to do so. After being informed about this phenomenon, 14 participants were able to inhibit it, at least partially. The results of whole brain and ROI analyses support the fact that both deliberate and unconscious imitations are based on similar neural mechanisms and networks, involving regions of the dorsal stream, during both perception and production steps of the imitation process. While no significant difference in brain activation was found between unconscious and deliberate imitations, the degree of imitation, however, appears to be determined by processes occurring during the perception step. Four regions of the dorsal stream: bilateral auditory cortex, bilateral supramarginal gyrus (SMG), and left Wernicke's area, indeed showed an activity that correlated significantly with the degree of imitation during the perception step.

## Introduction

When they interact, speakers tend to imitate their interlocutor's posture (Shockley et al., 2003), gestures, facial expressions, and breathing (Chartrand and Bargh, 1999; Estow and Jamieson, 2007; Sato and Yoshikawa, 2007). Regarding their interlocutor's speech, such convergence effects also occur at the phonetic, lexical, and syntactic levels (Natale, 1975; Pardo, 2006; Delvaux and Soquet, 2007; Kappes et al., 2009; Aubanel and Nguyen, 2010; Bailly and Lelong, 2010; Miller et al., 2010; Babel, 2012; Babel and Bulatov, 2012). The phenomenon of “phonetic convergence,” also referred to as “accommodation,” “entrainment,” “alignment,” or “chameleon effect,” not only concerns supra-segmental parameters such as vocal intensity (Natale, 1975), fundamental frequency (*f*_0_) (Gregory et al., 1993, 2000; Bosshardt et al., 1997; Goldinger, 1997; Babel and Bulatov, 2012) and long-term average spectrum (Gregory et al., 1993, 1997, 2000; Gregory and Webster, 1996) but also temporal and spectral cues to phonemes like voice onset time (VOT) of stop consonants (Sancier and Fowler, 1997; Shockley et al., 2004; Nielsen, 2011) and the first two formants of vowels (*F*_1_, *F*_2_; (Babel and Bulatov, 2012; Pardo, 2010; Sato et al., 2013). This phenomenon appears to be quite subtle, *f*_0_ and speech rate showing the greatest sensitivity to phonetic convergence (Pardo, 2010; Sato et al., 2013).

Most of the literature considers this convergence phenomenon as primarily driven by social or communicative motivations. Convergence behaviors may aim at placing the interaction on a “common ground” of sounds and gestures, which is hypothesized to improve communication at the social level and/or at the intelligibility level.

Several theories predict that speakers converge more toward people they like, and from whom they want to be liked in return (Byrne, 1997; Chartrand and Bargh, 1999; Babel, 2009), toward people they are acquainted with (Lelong and Bailly, 2012) or toward people who exert a leadership (Pardo, 2006) or any kind of social dominance/hierarchy on them (Gregory and Hoyt, 1982; Street and Giles, 1982; Gregory, 1986; Giles et al., 1991; Gregory and Webster, 1996). More generally, the Communication Accommodation Theory (CAT; Giles et al., 1991) considers phonetic convergence and divergence as a social tool to mark the desire to belong to or to distinguish oneself from a social group (Giles et al., 1973; Giles, 1973; Bourhis and Giles, 1977; Tajfel and Turner, 1979; Giles et al., 1991). Work by Krashen (1981) and Pardo (2006) supports the idea that phonetic convergence is driven by empathy, rather than by the desire to be liked. In any case, no evidence has been provided yet, supporting the idea that we like people more if they converge toward us [although, on the other side, previous studies showed that we like people more after imitating them (Adank et al., 2013)].

Phonetic convergence is also believed to improve communication at the intelligibility level. Producing speech sounds and lexical forms that are more similar to the own repertoire of the interlocutor may facilitate phonetic decoding and lexical access. However, no study has shown such intelligibility benefits yet [although, on the other side, previous studies showed it is easier to understand an accent after imitating it (Adank et al., 2010)]. In fact, this idea appears contradicted by the fact that our own speech—that cannot be more similar to our own sound repertoire—is not more intelligible to us than speech produced by others (Hawks, 1985).

Several additional observations lead us to partly reconsider the idea that phonetic convergence may be primarily driven by social and communicative motivations. First, phonetic convergence was also observed in non-interactive tasks of speech production (Goldinger, 1998; Namy et al., 2002; Shockley et al., 2004; Vallabha and Tuller, 2004), even at the basic level of vowel production (Sato et al., 2013). Partial imitation of lip gestures and vocal sounds was also observed in newborns and small children (Heimann et al., 1989; Meltzoff and Moore, 1997). Such imitation processes appear to be involuntary (Garrod and Clark, 1993) and are believed to play a key role in cognitive development, in particular for language acquisition (Chen et al., 2004; Serkhane et al., 2005; Nagy, 2006). Some authors support the idea that these automatic imitation processes still exist in adults, but that they may be neutralized by inhibition processes. They formulate this hypothesis from the observation of patients with fontal brain lesions and a loss of social inhibition, who systematically repeat and imitate their interlocutor (Brass et al., 2003, 2005; Spengler et al., 2010). These studies suggest that imitation would be innate and involuntary while inhibiting imitation, and controlling the degree of this inhibition, is what we may learn with age.

Rizzolatti and colleagues (e.g., Iacoboni et al., 1999; Rizzolatti et al., 2001; Gallese, 2003) have suggested the idea of a “direct matching” between perception and action, as the basis for imitation of motor tasks. Main empirical support of this theoretical proposal comes from the discovery of mirror neurons in the macaque brain (Rizzolatti et al., 1996, 2002). Mirror neurons are a small subset of neurons, found in the macaque ventral premotor cortex and the anterior inferior parietal lobule that fire both during the production of goal-directed actions and during the observation of a similar action made by another individual. In humans, homologous brain regions were also found to be involved in both action perception and production (notably, the pars opercularis of Broca's area, located in the posterior part of the inferior frontal gyrus; Rizzolatti and Arbib, 1998). Such a “motor resonance” was observed not only for finger, hand and arm movements (Tanaka and Inui, 2002; Buccino et al., 2004; Molnar-Szakacs et al., 2005), but also for mouth and lip movements (Fadiga et al., 2002; Wilson, 2004; Skipper et al., 2007; D'Ausilio et al., 2011). This overlapping network appears to be hard wired, or at least to function from the very beginning of life (Sommerville et al., 2005; Nyström, 2008).

Regarding speech, a number of models also support the idea of a direct matching between perception and motor systems (for reviews, see Galantucci et al., 2006; Schwartz et al., 2012). Motor theories of speech perception argue that speech is primarily perceived as articulatory gestures (Liberman and Mattingly, 1985; Fowler, 1986) and sensory-motor theories postulate that phonetic coding/decoding and representations are shared by speech production and perception systems (Skipper et al., 2007; Rauschecker and Scott, 2009; Schwartz et al., 2012). Brain imaging studies provide evidence for an involvement of the motor system in speech perception (Fadiga et al., 2002; Wilson, 2004; Skipper et al., 2007). Anatomical connections between posterior superior temporal regions, the inferior parietal lobule, and the posterior ventrolateral frontal lobe in the premotor cortex were attested using diffusion tensor magnetic resonance imaging (Catani and Jones, 2005). Recent neurobiological models of speech perception and production postulate the existence of a dorsal sensory-motor stream (Hickok and Poeppel, 2000, 2004, 2007; Poeppel and Hickok, 2004) mapping acoustic representations onto articulatory representations, including the posterior inferior frontal gyrus, the premotor cortex, the posterior superior temporal gyrus/sulcus, and the inferior parietal lobule (Callan et al., 2004; Guenther, 2006; Skipper et al., 2007; Dick et al., 2010).

To sum up, all these observations and models support the idea that humans have shared representations of the motor commands of an action and of its sensory consequences. This functional coupling between perception and action systems, through these shared representations, argues for perception not only consisting in information decoding but also contributing to the automatic and involuntary “update” or “recalibration” of these shared sensory-motor representations.

This brings us to reconsider the mechanisms underlying the phenomenon of phonetic convergence and to explore the hypothesis that automatic and involuntary imitation of phonetic features might reflect a sensory-motor learning, taking place as soon as speech is perceived. In favor of this hypothesis is the fact that speakers modify their way of speaking not only during the interaction with their interlocutor, but also after the interaction. This “after-effect” concerns not only speech production, but also speech perception: vowel categorization was found to be modified after repeated exposure to someone else's speech (Sato et al., 2013). Furthermore, passive listening, without any motor involvement, appears to be sufficient to observe these after-effects (Sato et al., 2013).

The present study aimed at determining the neural substrates of phonetic convergence and more particularly at: (1) understanding whether phonetic convergence and deliberate imitation of speech are underpinned by the same neurocognitive mechanisms, (2) examining to what extent sensory-motor brain areas are involved during deliberate and unconscious imitations of speech, and (3) better understanding the degree of control and consciousness that one can have on imitation and its inhibition.

On the basis of previous studies, showing the involvement of the dorsal stream in voluntary imitation of speech (Damasio and Damasio, 1980; Caramazza et al., 1981; Bartha and Benke, 2003; Molenberghs et al., 2009; Irwin et al., 2011; Reiterer et al., 2011) and fast overt repetition (Peschke et al., 2009), we assumed the dorsal stream to be also involved in phonetic convergence. We expected deliberate imitation and unconscious convergence to be based on the same mechanisms but to rely on the modulated activation of the dorsal stream, particularly during the perception step of the perception-action loop. Finally, we also hypothesized that phonetic convergence can be inhibited to some extent, and that this inhibition also relies on activity changes of the dorsal stream.

To explore these hypotheses, we simultaneously recorded speech signals and neural responses of 16 participants, in three tasks of speech imitation with varying degrees of will and consciousness: voluntary imitation, phonetic convergence, intended inhibition of phonetic convergence. In these tasks, we focused on one phonetic feature particularly sensitive to that phenomenon: *f*_0_, which was varied specifically for each participant, from −20 to +20% around his/her own average pitch. We used a go-nogo paradigm in order to compare brain activations during the whole imitative process or during its perception and production steps only. In addition, two other speech control tasks (passive perception and production) were included in order to compare brain activations during perception and motor steps of the imitative process, with brain activations during usual perception and non-imitative production of vowels.

## Methods

### Participants

Sixteen right-handed and healthy participants (11 males and 4 females of 27 ± 5 years old), French native speakers, volunteered to participate in the experiment. None of them had any speaking or hearing disorders. None of them had previously received explicit information about phonetic convergence phenomena. The study received the ethic approval from the Centre Hospitalier Universitaire de Grenoble, from the Comité de Protection des Personnes pour la Recherche Biomédicale de Grenoble and from the Agence Française de Sécurité Sanitaire des Produits.

### Procedure

The experiment consisted of three tasks of interest and two reference tasks.

T1. Reference task: passive auditory perception of vowels.
T2. Vowel production task. The vowels to be produced were played to the participant through headphones. Participants were expected to partly and unconsciously imitate these stimuli (convergence effect).
T3. Vowel production reference task. The vowels to be produced were displayed on a screen viewed by the participant. Participants were expected to produce vowels according to their own speech representations.
T4. Vowel imitation task. Like in T2, vowels were played to the participant through headphones. Participants were asked to produce these vowels and to « imitate the voice heard ».
T5. Vowel production and convergence inhibition task. Participants were briefly informed about the existence of convergence phenomena. Like in T2 and T3, vowels were played to the participant through headphones. They were asked to produce these vowels as close as they could from their habitual production, trying not to follow the stimuli.

Participants were simply informed that the experiment would consist in the production and perception of vowels. The two first tasks were presented as such to the participants, in order for them not to suspect the audio stimuli to influence their own production. The voluntary imitation and inhibition tasks were thus left for the end of the experiment. These five tasks were followed by a brain anatomical scan. The whole procedure was completed in one and an half hour.

The audio stimuli used in the conditions T1, T2, T4, and T5 consisted of 27 different vowels, specifically selected for each participant. First, a vowel database with modified pitches was created from 3 French vowels ([e], [oe], [o]) produced by a reference male speaker and a reference female speaker. Pitches were artificially shifted by steps of 5 Hz from 80 to 180 Hz for the male vowels, and from 150 to 350 Hz for the female vowels. This pitch manipulation was performed using the PSOLA module integrated in Praat, which enables to modify pitch without affecting formants or speech rate. Before the experiment, each participant was also recorded while producing a series of vowels, in order to determine his/her habitual pitch (see Table 1). Finally, for each participant, 27 stimuli were selected from the vowel database, with the 9 quantified frequencies closest to 80, 85, 90, 95, 100, 105, 110, 115, and 120% of his/her habitual pitch. The visual stimuli used in the condition T3 consisted of the 3 symbols «éé», «eu», and «oo».

**Table 1:** **Participants' information**.

	**S1**	**S2**	**S3**	**S4**	**S5**	**S6**	**S7**	**S8**	**S9**	**S10**	**S11**	**S12**	**S13**	**S14**	**S15**	**S16**
Gender	M	F	F	M	M	M	M	F	M	F	M	M	M	M	F	M
Age	22	30	27	23	25	39	24	30	24	25	38	28	25	27	27	23
Average habitual *f*_0_ (Hz)	129 ± 3	219 ± 6	198± 5	127 ± 3	111 ± 1	130 ± 5	131 ± 3	298 ± 6	131 ± 5	239 ± 12	125 ± 6	135 ± 9	126 ± 4	146 ± 4	237 ± 18	121 ± 8

The two reference tasks (T1 and T3) consisted in 54 trials. The 27 audio or visual stimuli described above were presented in a pseudo-random order and in alternation with 27 « void » stimuli (i.e., no sound in T1 or no displayed vowel in T3). These « void » stimuli were used as a baseline for the comparison of neural activations. Each trial lasted 10 s. Stimuli were played (T1) or displayed (T3) during the first 500 ms. One second later, a fixation cross was displayed during 500 ms which indicated when the participant had to produce the vowel for the T3 condition.

The three tasks of interest (T2, T4, and T5) consisted of 81 trials. In these tasks, the 27 audio stimuli were presented twice, in a pseudo-random order and in alternation with the 27 « void » stimuli (i.e., no sound). Concretely, one third of the time, audio stimuli were followed by a green cross, indicating to the participant that he/she should produce the vowel (« Go »). One other third of the time, audio stimuli were followed by a red cross, meaning that the participant should remain quiet (« No Go »). The last third of the time, no stimulus was played and a red cross was displayed (Baseline). This go/no-go paradigm enables to compare the neural activations in a double task of speech production and perception, with those in a task of « active » perception, i.e., when participants perceive vowels with the goal of producing them afterwards, but finally without carrying out any motor action.

### Material and data acquisition

Visual instructions were displayed on a screen located behind the participant, using a video projector and the Presentation software (Neurobehavioral Systems, Albany, EU). Participants could read them by reflection, thanks to a mirror placed above their eyes. Audio stimuli were played though MRI-compatible headphones. The audio level was set to a sufficient intensity so that participants could hear the stimuli correctly, despite the earplugs they wore to protect them from the scanner noise. The production of vowels was recorded thanks to a microphone placed 1 m away from their mouth.

Anatomic and functional images were acquired with a whole body 3T scanner (Bruker MedSpec S300) equipped with a transmit/receive quadrature volume head coil. The fMRI experiment consisted of five functional runs and one anatomical run. Functional images were obtained using a T2^*^-weighted, echoplanar imaging (EPI) sequence with whole-brain coverage (*TR* = 10 s, acquisition time = 2600 ms, *TE* = 30 ms, flip angle = 90°). Each functional scan comprised forty axial slices parallel to the anteroposterior commissural plane acquired in interleaved order (72 × 72 matrix; field of view: 216 × 216 mm^2^; 3 × 3 mm^2^ in plane resolution with a slice thickness of 3 mm without gap). A « sparse sampling » acquisition paradigm was used in order to minimize potential artifacts articulatory movements could induce on functional images. This acquisition technique stems from the time delay existing between the neural activity linked to a motor or perceptual task and the associated hemodynamic response. Based on the estimation of this delay in tasks of speech production and perception by previous studies (Grabski et al., 2013), the functional scan was chosen to start 4.7 s after stimulus perception, thus 3.7 s after vowel production (in T2–T5). A high-resolution T1-weighted whole-brain structural image was acquired for each participant after the third functional run (MP-RAGE, sagittal volume of 256 × 224 × 176 mm^3^ with a 1 mm isotropic resolution, inversion time = 900 ms, two segments, segment repetition time = 2500 ms, segment duration = 1795 ms, *TR*/*TE* = 16/5 in ms with 35% partial echo, flip angle = 8°).

### Acoustic analysis

The acoustic analyses were performed using Praat software. A semi-automatic procedure was used to segment vowels on the basis of intensity and duration criteria. Hesitations and mispronunciations were removed from the analyses. *f*_0_ values were calculated, using an autocorrelation method, from a time interval defined as ±25 ms of the maximum peak intensity of the sound file.

The stimuli were specific to each participant, with *f*_0_ values varying between approximately −20 and +20% of the participant's average habitual *f*_0_ (see Table 1). Consequently, the *f*_0_ values of the produced vowels were also converted to the participant's range, expressed as the percentage of deviation from his/her average habitual *f*_0_: Δ *f*_0_ = (*f*_0produced_ − *f*_0habitual_)/*f*_0habitual_.

### fMRI data preprocessing and statistical analysis

Data were analyzed with the software SPM5 (Statistical Parametric Mapping, Wellcome Trust Centre for Neuroimaging, London, UK). The fMRI data of one participant (S3) were artifacted by a metalic pin and could therefore not be included in the analysis. The results reported in the fMRI data section of this article thus concern the 15 remaining participants.

For each participant, functional images were realigned, normalized in the reference space of the Montreal Neurological Institute (MNI) and smoothed with a 6 mm width Gaussian low-pass filter.

The hemodynamic responses corresponding to the experimental conditions were then estimated with a general linear model, including the characterization of a unique impulse response for each functional scan and taking body movements into account through regressors of non-interest.

#### Whole brain statistical analysis

Eight T-contrasts were tested (see Table 2), in order to identify brain regions specifically involved in vowel perception or production (when listening passively or actively, and with different degrees of imitation), compared to a resting condition.

**Table 2:**
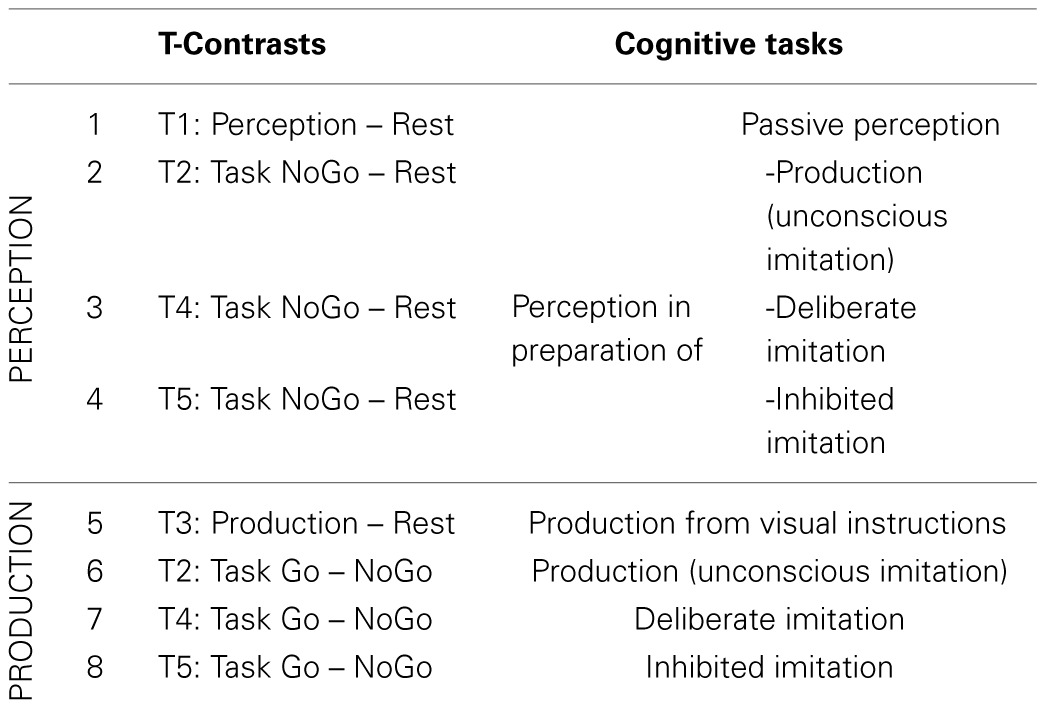
**Detail of the eight individual t-contrasts tested, and of the corresponding cognitive tasks explored**.

Using SPM, a flexible factorial group analysis was conducted from these individual contrasts, corresponding to a One-Way repeated measures ANOVA (one factor TASK with 8 levels).

Eight T-contrasts were tested in order to identify brain regions specifically involved in each task of vowel perception and/or production, compared to a resting condition. Two conjunctions were calculated from the first four contrasts examining neural correlates of vowel perception, as well as from the four following contrasts examining neural correlates of vowel production. Two F-contrasts tested the main effect between the vowel perception conditions (1,2,3,4) and between the vowel production conditions (5,6,7,8).

For these contrasts, statistical significance was considered for *p* < 0.05, corrected for multiple comparisons («false discovery rate » test for perception tasks and « family-wise error » test for production tasks), with activation clusters wider than 25 voxels.

The 3D coordinates of the center of gravity of the activated clusters, normalized in the MNI reference space were assigned to functional areas of the brain thanks to the SPM Anatomy toolbox and on the basis of cytoarchitechtonic probabilities. When not assigned in the SPM Anatomy toolbox, brain regions were labeled using Talairach Daemon (Lancaster et al., 2000).

#### Regions of interest analysis

This study hypothesizes that the dorsal stream would be involved in speech imitation and phonetic convergence. Particular attention was therefore paid to neural activations in regions of the dorsal stream. With the SPM Anatomy toolbox, 7 ROIs were defined in both hemispheres, from the cytoarchitechtonic probability of

– Region TE (including TE1.0, TE1.1, and TE1.2)
– Region TE3 (Wernicke's area, including the Spt area),
– Supramarginal Gyrus (IPC PF, PFm, PFcm)
– Region BA6 (premotor cortex and supplementary motor area)
– Regions BA44 and BA45 (Broca's area)
– The Insula

Using Marsbar, eight T-contrasts (similar to Table 2) were tested from individual fRMI data, in order to determine the difference of neural activations in the ROIs previously defined, between tasks of vowel perception and/or production (when listening passively or actively, and with different degrees of imitation), and a resting condition.

Using SPSS software, a One-Way repeated measures ANOVA was then conducted on these individual differences of neural activation observed in each ROI. Statistical significance was considerered for *p* < 0.001, *post-hoc* analyses being corrected for multiple comparisons (Bonferroni).

Finally, we performed a Pearson correlation analysis to determine the correlation between the average activation of each ROI, for each participant, in the deliberate and unconscious imitation tasks, and their demonstrated degree of imitation (defined from the behavioral data, as the slope coefficient between their produced *f*_0_, and that of the followed stimuli).

## Results

### Behavioral results

**Figure 1:**
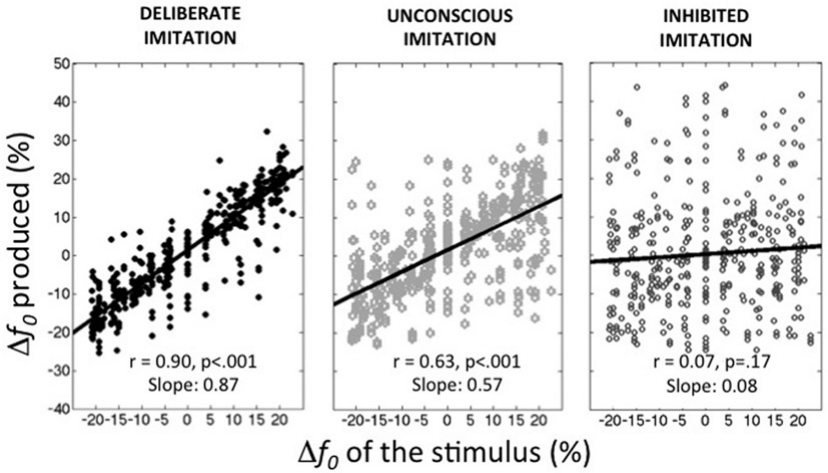
**Average behaviors observed in the three tasks of deliberate, unconscious, and inhibited imitation**. Vowel stimuli were presented with 9 *f*_0_ values, varying around the habitual average pitch of each participant. The y-axis represents how participants modified their produced *f*_0_ from their habitual average *f*_0_ (see Table 1).

Figure 1 summarizes the average behaviors observed in the deliberate imitation (T3), unconscious imitation (T2), and inhibited imitation (T5) tasks. On average, the observed tendencies confirmed our expectations:

– participants were able to imitate almost perfectly the pitch of the audio stimuli (T3; slope coefficient of 0.87, *r* = 0.900, *p* < 0.001).
– participants unconsciously followed the pitch of the audio stimuli in the production task when vowels were presented auditorily (T2; *r* = 0.635, *p* < 0.001). This unconscious imitation was, however, not as strong as voluntary imitation (Slope coefficient of 0.57). It is worthwhile noting that this order of magnitude is much higher than the convergence effects usually reported in behavioral studies (slope coefficient of 0.08 in Sato et al., 2013, for instance).
– participants were able to inhibit almost completely this convergence effect when informed about its existence (T5; Slope coefficient of 0.08, *r* = 0.067, *p* = 0.17).

**Figure 2:**
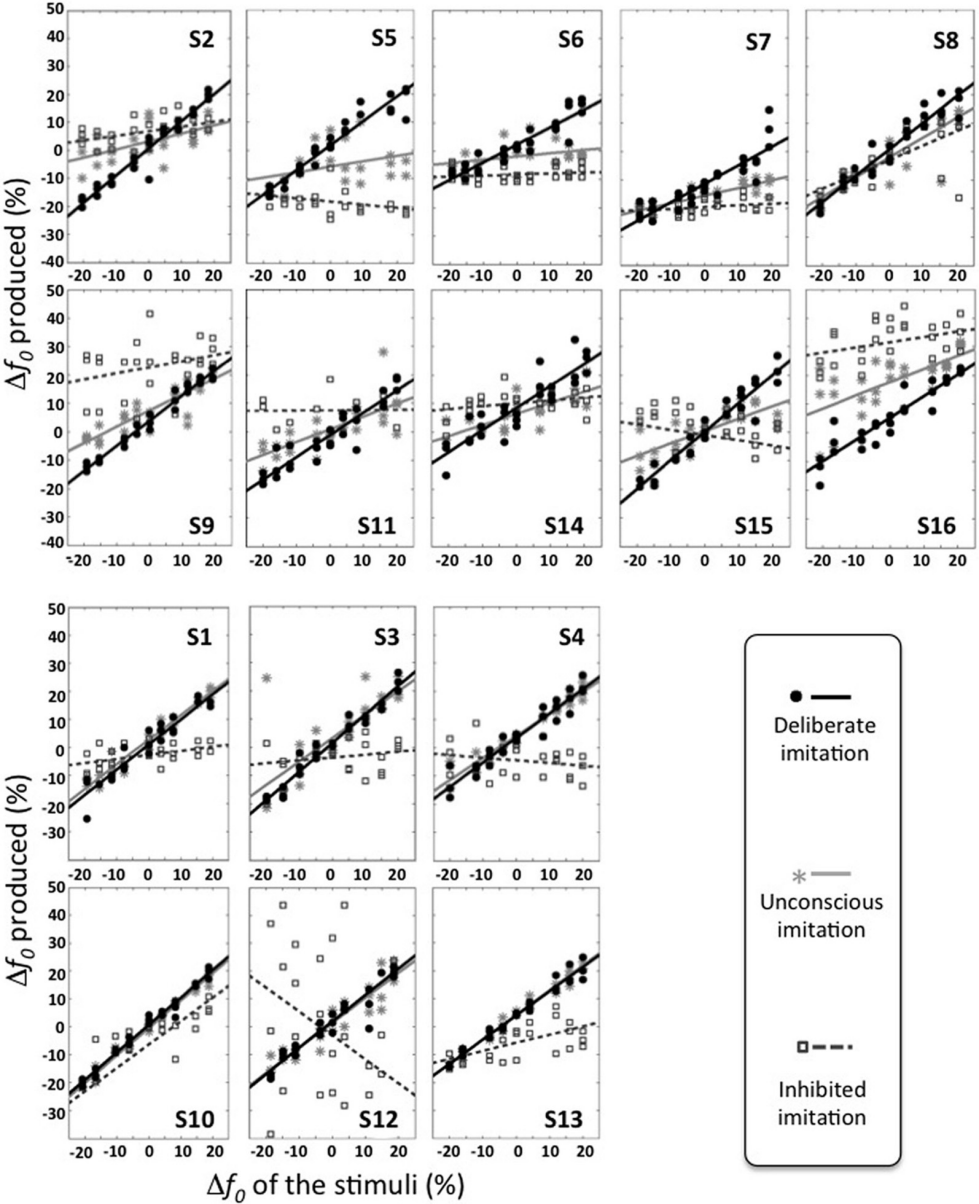
**Individual behaviors observed in the three tasks of deliberate, unconscious, and inhibited imitation**. Vowel stimuli were presented with 9 *f*_0_ values, varying around the habitual average pitch of each participant. The y axis represents how participants modified their produced *f*_0_ from their habitual average *f*_0_. The six participants of the bottom panel did not show significant difference in their behavior between the tasks of deliberate and unconscious imitation.

At the individual level, however, varying behaviors were observed. Figure 2 gives an overview of these different individual behaviors. Table 3 summarizes the results of the Pearson correlation analysis.

**Table 3:** **Individual results of Pearson's correlation test, examining the degree of imitation in the tasks of deliberate, unconscious, and inhibited imitation**.

	**Deliberate imitation**			**Unconscious imitation**			**Inhibited imitation**		
	**Slope**	***r***	***p***	**Slope**	***r***	***p***	**Slope**	***r***	***p***
S1	0.90	0.96	<0.001	0.88	0.98	<0.001	0.15	0.52	=0.005
S2	0.98	0.98	<0.001	0.29	0.59	=0.001	0.16	0.48	=0.012
S3	1.00	0.98	<0.001	0.83	0.77	<0.001	0.10	0.22	=0.26
S4	0.87	0.96	<0.001	0.78	0.97	<0.001	−0.09	−0.21	=0.285
S5	0.87	0.95	<0.001	0.19	0.36	=0.005	−0.11	−036	=0.069
S6	0.62	0.93	<0.001	0.11	0.38	=0.005	0.03	0.19	=0.336
S7	0.65	0.89	<0.001	0.27	0.75	<0.001	0.06	0.32	=0.099
S8	0.93	0.96	<0.001	0.69	0.86	<0.001	0.51	0.70	<0.001
S9	0.89	0.99	<0.001	0.57	0.88	<0.001	0.22	0.31	=0.112
S10	0.98	0.99	<0.001	0.98	0.99	<0.001	0.65	0.85	<0.001
S11	0.78	0.93	<0.001	0.45	0.74	<0.001	0.01	0.02	=0.952
S12	0.94	0.96	<0.001	0.89	0.94	<0.001	−0.86	−0.40	=0.039
S13	0.87	0.99	<0.001	0.88	0.99	<0.001	0.29	0.61	<0.001
S14	0.78	0.90	<0.001	0.39	0.80	<0.001	0.10	0.35	=0.078
S15	1.00	0.96	<0.001	0.44	0.77	<0.001	−0.20	−0.50	=0.009
S16	0.75	0.93	<0.001	0.46	0.66	<0.001	0.18	0.35	=0.076

Results that are statistically significant (*p* < *0.05*) are indicated in black and non significant results are indicated in grey.

Some participants demonstrated better imitation abilities than others but all of them were able to follow variations of pitch (slope coefficients from 0.62 to 1.00).

Five speakers (see bottom panel of Figure 2) did not show any significant behavioral difference in the variation of *f*_0_ between deliberate and unconscious imitations: they completely followed the pitch of the audio stimuli, even in task T3 for which they were not told of or conscious about convergence effects (slope coefficients from 0.78 to 0.98).

Eight speakers (see top panel of Figure 2) showed a significantly weaker degree of imitation in the unconscious imitation task. The slope of the convergence effect showed a great inter-speaker variability, from 0.11 to 0.69.

Great inter-speaker variability was observed in the inhibition task too. Ten out of 16 speakers (S3, S4, S5, S6, S7, S9, S11, S12, S14, S16) did not show a significant correlation between their produced *f*_0_ and that of the stimuli in this task, which supports the idea that they were able to inhibit the convergence effect.

Three speakers (S1, S2, S13) showed a significant and positive correlation between their produced *f*_0_ and that of the stimuli, with a significantly weaker linear regression slope than in the unconscious imitation task (respectively 0.15, 0.16, and 0.29). These speakers were thus able to partially compensate for the convergence effect.

Two speakers (S8 and S10) also showed a significant correlation between their produced *f*_0_ and that of the stimuli, but with a linear regression slope significantly almost as high (respectively 0.51 and 0.65) as in the unconscious imitation task. Inhibiting the convergence effect was therefore very hard for these participants.

Finally, one of the speakers (S15) even showed a significant but negative correlation between her produced *f*_0_ and that of the stimuli (slope coefficient of −0.20, *r* = −0.500, *p* = 0.009), indicating a strategy of overcompensation of the convergence effect.

### Neural activations

#### Systems of speech perception and production

**Figure 3:**
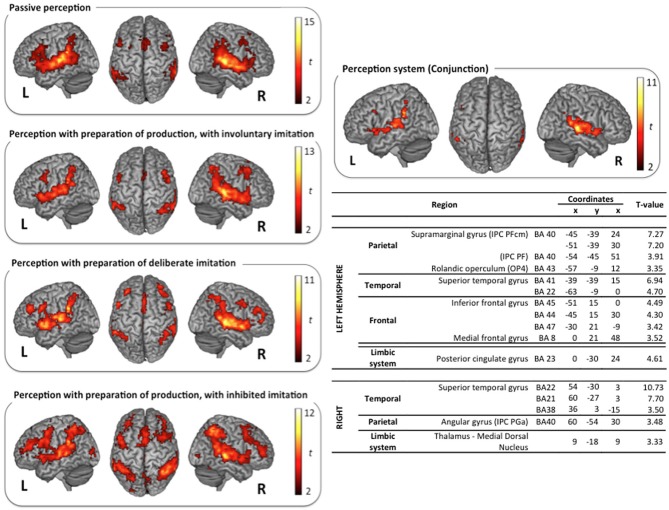
**Surface rendering of brain regions activated in the perception tasks and maximum activation peak summary for their conjunction**. All contrasts are computed from the random-effect group analysis (*p* < 0.05, FDR corrected, cluster extent threshold of 25 voxels, coordinates in MNI space).

**Figure 4:**
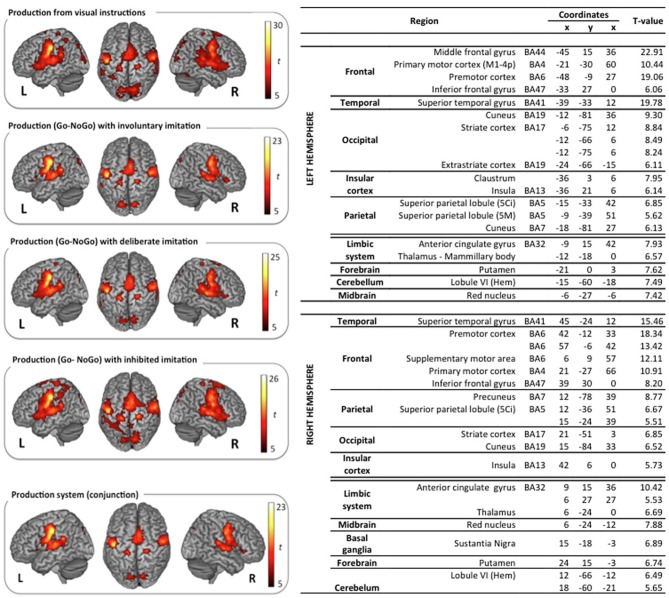
**Surface rendering of brain regions activated in the production tasks and maximum activation peak summary for their conjunction**. All contrasts are computed from the random-effect group analysis (*p* < 0.05, FWE corrected, cluster extent threshold of 25 voxels, coordinates in MNI space).

The classical neural networks for speech perception and production were observed in the reference tasks of passive vowel perception and vowel production from visual instructions. Surface rendering of brain activity observed in these reference tasks is displayed in the top left panels of (Figures 3, 4).

***Vowel perception and production reference tasks***. Passive vowel perception induced large bilateral activation of the superior temporal gyrus (STG), from its anterior part to the temporo-parietal junction. Maximum activity was displayed in the primary, secondary, and associative auditory cortices, extending to the middle temporal gyrus (MTG), the Insula, and the rolandic operculum. Bilateral activations were also observed in the inferior frontal gyrus (IFG), within the pars opercularis and triangularis, extending ventrally to the pars orbitalis in the left hemisphere, and rostrodorsally to BA46 in the right hemisphere. Additional frontal activations were observed bilaterally in the dorsolateral prefrontal cortex, the premotor cortex, and the supplementary motor area (SMA). Superior and inferior parietal activations were observed bilaterally in the supramarginal gyrus (SMG), the rolandic operculum, and in the left angular gyrus. Further activity was displayed in limbic structures, in particular in the thalamus and the cingulate cortex (anterior part in the left hemisphere and middle part in the right hemisphere), and in the basal ganglia (right caudate nucleus).

Vowel production from visual instructions induced bilateral activations of the premotor, primary motor, and sensorimotor cortices, and of the SMA. Bilateral activations were also observed in the IFG (pars opercularis and triangularis) and in the STG, extending to the rolandic operculum and the SMG. Additional activations were displayed bilaterally in superior and posterior parts of the parietal cortex, including the precuneus, the associative cortex, and the angular gyrus. Further activity was found in the left inferior temporal gyrus, and bilaterally in the cerebellum, the cingulate cortex (anterior and middle part in the left hemisphere, middle part only in the right hemisphere), and the visual cortex.

***Speech perception and production with various types and degrees of imitation***. The typical neural network for speech perception was found again in the three other tasks of active perception, in preparation of deliberate, unconscious, or inhibited imitations (NoGo trials). Surface rendering of the conjunction of the brain activity observed in all the perception tasks is displayed in the right panel of Figure 3, with a summary of the maximum activation peaks.

This shared perception network involves bilateral activation of the STG, extending to the rolandic operculum and to the left Insula. Frontal regions participate in this network in the left hemisphere only, in particular Broca's area (pars opercularis and triangularis of the IFG), and the frontal region BA8. It also involves inferior parietal regions in both hemispheres: the SMG, extending to the rolandic operculum on the left side, and the angular gyrus on the right side. Further shared activations were found in the limbic system (right thalamus and left posterior cingulate cortex). A significant activation of the dorsolateral prefrontal region BA46 was also observed during the perception step of deliberate and inhibited imitations (NoGo trials, see Figure 1). However, the activity of this region was not found to vary significantly between the 4 different speech perception tasks (see paragraph Whole Brain Analysis below).

Similarly, the typical network for speech production was also observed in the three other speech production tasks with deliberate, unconscious, or inhibited imitations (t-contrast between the Go and the NoGo trials). Surface rendering of the conjunction of the brain activity observed in all the perception tasks is displayed in the right panel of Figure 4, with a summary of the maximum activation peaks.

This shared production network involves bilateral activations in the premotor, primary motor and sensorimotor cortices, extending to the IFG (pars triangularis) and to the SMA. It also involves the primary auditory cortex in the STG, extending to the rolandic operculum, and to the Insula. Further shared activations were found in posterior parietal regions, including the precuneus and the associative cortex, as well as in the limbic system (anterior cingulate gyrus, thalamus), the cerebellum, the putamen, the red nucleus, and the right basal ganglia (substantia nigra).

#### Comparison of deliberate, unconscious and inhibited imitations

***Whole brain analysis***. Using a corrected statistical analysis, no brain region was found to be significantly more or less activated between the four speech perception tasks.

No brain region was found to be significantly modulated in activation between the four speech production tasks either.

***ROI analysis: differences between tasks***. Tables 4, 5 summarize the results of further analysis and comparison of brain activity, more specifically in regions of interest of the dorsal stream.

**Table 4:**
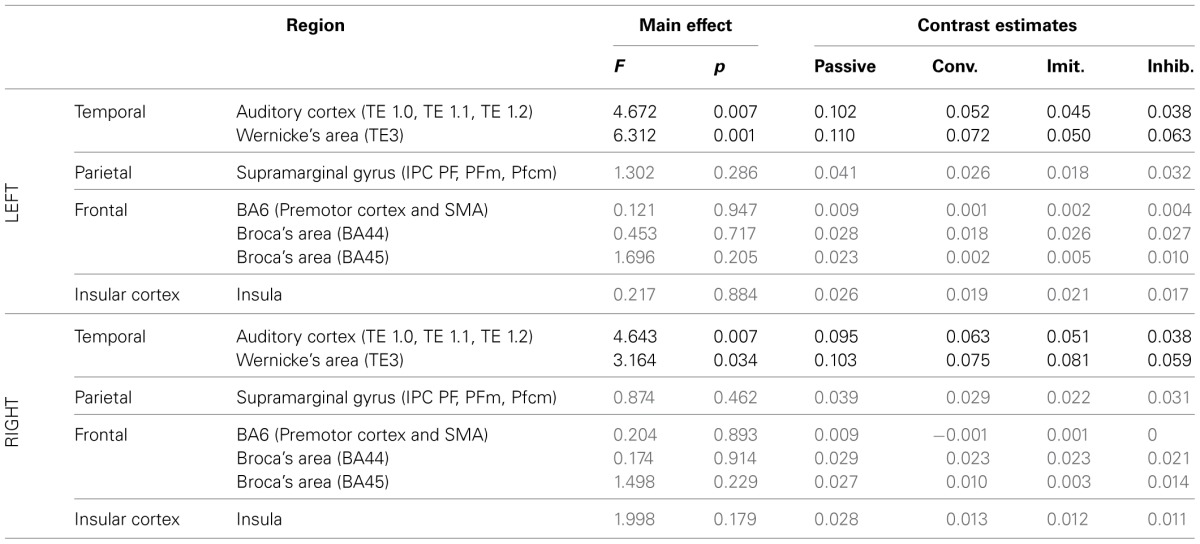
**Results of the ROI analysis, comparing brain activity in several regions of the dorsal stream, between the four speech perception tasks [passive perception, perception in preparation of vowel production (phonetic convergence), of deliberate imitation, or inhibited imitation]**.

Results that are statistically significant (*p* < *0.05*) are indicated in black and non significant results are indicated in grey.

**Table 5:**
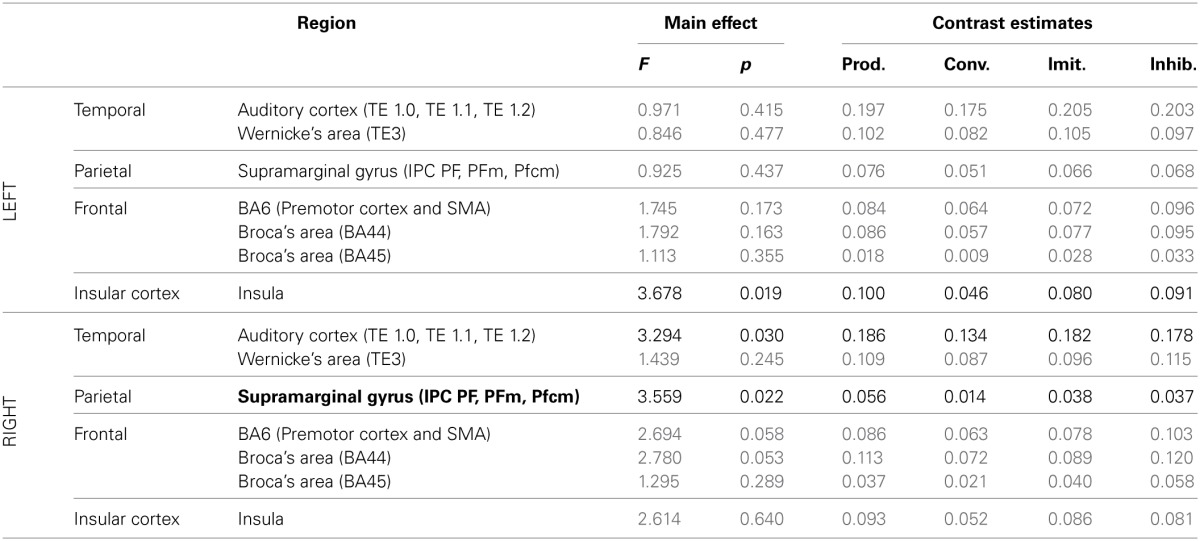
**Results of the ROI analysis, comparing brain activity in several regions of the dorsal stream, between the four speech production tasks (production from visual instructions, production with phonetic convergence, deliberate imitation, inhibited imitation)**.

Results that are statistically significant (*p* < *0.05*) are indicated in black and non significant results are indicated in grey. Corresponding ROIs are indicated in bold.

The ROI analysis showed a significant modulation of brain activity in the auditory cortex and Wernicke's area, bilaterally, between the four vowel perception tasks with varying degrees and types of imitation. No tendency was observed toward increasing or decreasing activation with the degree of imitation.

For the production tasks, the ROI analysis again highlighted the right auditory cortex as a brain region of the dorsal stream whose activity is significantly modulated between the four vowel production tasks with varying degrees and types of imitation. The left Insula and the right SMG were two additional regions of the dorsal stream that demonstrated a significant modulation of their neural activation. No tendency was found toward increasing or decreasing activation of these regions with the degree of imitation.

***ROI analysis: correlations with behavioral data***. Table 6 summarizes the results of Pearson's correlation analysis that examined the correlation between brain activity in regions of interest of the dorsal stream and the degree of imitation in the deliberate and unconscious imitation tasks. The degree of imitation was evaluated from the slope of the variation in the produced *f*_0_, as a function of the *f*_0_ of the stimuli.

**Table 6:**
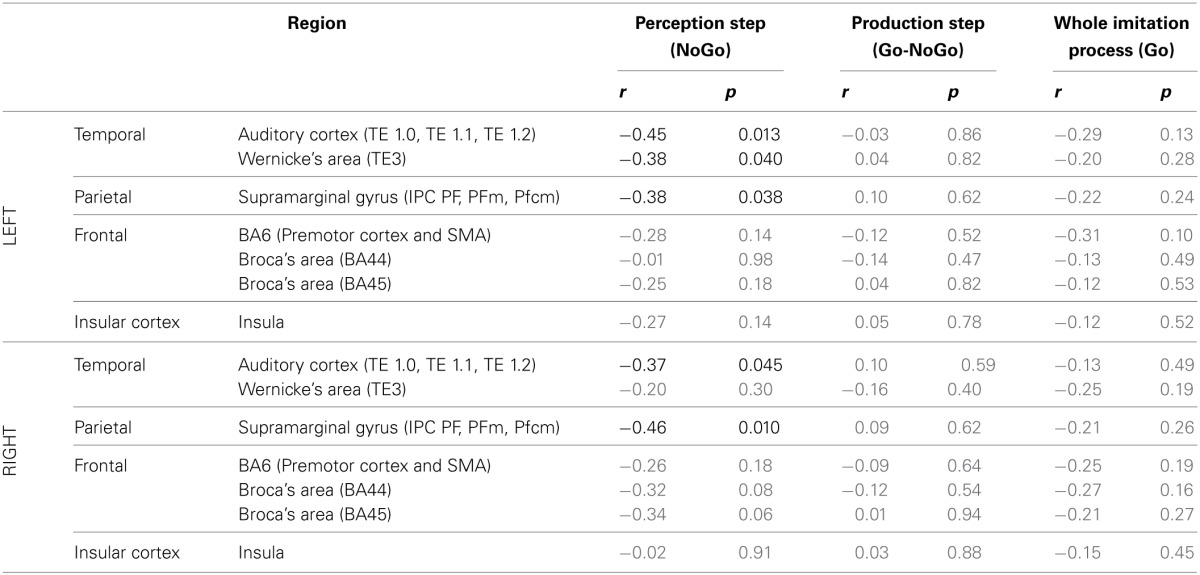
**Results of Pearson's correlation analysis, examining the correlation between the degree of imitation during the tasks of deliberate and unconscious imitation, and the brain activity in several regions of the dorsal stream, during the whole imitative process (Go trials), during the perception step only (NoGo trials) and during the production step only (Go-Nogo contrast)**.

Results that are statistically significant (*p* < *0.05*) are indicated in black and non significant results are indicated in grey.

Again, in the two active perception tasks, preparing a deliberate or unconscious imitation, brain activity in both left and right auditory cortices was found to correlate significantly with the degree of following imitation. So did left Wernicke's area and the SMG, bilaterally.

On the other hand, no brain region was found to vary in activation with a significant correlation with the degree of imitation, for the production step of the deliberate and unconscious imitation only tasks (Go-NoGo) or for the entire process of these tasks (Go).

## Discussion

In line with previous studies on phonetic convergence, our results show that speakers follow and unconsciously imitate the phonetic features (here *f*_0_) of voices they are exposed to, even when unaware of this imitation phenomenon and without being instructed to do so. Our results, however, differ from previous studies by a much greater order of magnitude in the degree of unconscious imitation. Indeed, slope coefficients in unconscious imitation of *f*_0_ ranged from 0.11 to 0.99 in our study (0.57 on average), whereas previous studies rather reported slope coefficients of 0.08 (Sato et al., 2013). Participants were debriefed after the experiment and they all said that they had not inferred that the study dealt with imitation, before accomplishing the deliberate imitation task (T4), and that they had neither thought nor tried to imitate the stimuli during the second vowel production task (T2). This discrepancy between our results and those of previous studies cannot be interpreted in terms of interactive vs. non-interactive protocols, as Sato et al. (2013) also used non-interactive tasks of vowel perception and production, similar to this study. One possible explanation is that such a high degree of unconscious imitation may come from the vowel production task, which may be closer to singing than to speech. Another explanation is that we used participant-specific stimuli in this experiment, varying in *f*_0_ from −20% to +20% of each participant's average habitual pitch. The pitch of these stimuli was consequently closer to the own pitch of each participant than in other studies on phonetic convergence, where the same stimuli are used for all the participants. One may suggest that phonetic convergence is not a linear phenomenon and that speakers are more disposed to completely mimic a voice already similar to their own voice, because the target is reachable and does not require much more effort to produce than their usual intra-individual variations. On the contrary, speakers may demonstrate a lesser degree of unconscious imitation toward voices that are very different from their own, because the target is out of reach or would induce vocal discomfort. Supporting this idea, one participant of this experiment (S5) was found to follow completely the pitch of the stimuli for values below 5% of his average habitual pitch (slope of 1.0). Above that pitch height, he did not follow the stimuli further and “saturated” to a constant *f*_0_ value (see Figure 2). Another argument comes from studies of intra-individual variations in habitual pitch, which is reported to vary as much as plus or minus three semitones, i.e., ~18% (Coleman and Markham, 1991). We can thus infer that the participants in our study have not made any particular effort to imitate the stimuli they were exposed to, which may explain why the degree of imitation is much greater than in the literature.

As in previous studies, we also observed a great inter-individual variability in the degree of deliberate imitation, with slope coefficients observed ranging from 0.62 to 1.00. Such a result is consistent with Pfordresher and Brown (2007), showing that about 15% of the population fails to imitate the pitch of a song by more than a semitone. Great inter-individual variability was also observed in the degree of unconscious imitation. In particular, a group of six participants demonstrated a similar degree of imitation for deliberate and unconscious imitation (slope coefficients from 0.78 to 1.00) whereas the other participants showed slope coefficients ranging from 0.11 to 0.69. Babel and Bulatov (2012) also reported a substantial inter-speaker variability in *f*_0_ accommodation, with some participants even diverging from their interlocutor. Although it has been suggested that female talkers may be better imitators (Pardo, 2006), we could not relate the different imitation abilities to the participant's gender or to their level of musical training. It is more likely, as suggested by Postma and Nilsenova (2012) or Lewandowski (2009) that inter-individual differences in the ability to imitate *f*_0_ or a foreign accent (“phonetic talent”) may be related to the neurocognitive capacity to extract acoustic parameters (pitch, in particular) from the speech signal. Supporting this idea, in this study we found a significant correlation between the degree of imitation and brain activity in the auditory cortex, while the lateral Heschl gyrus is consensually considered as the “pitch processing center” (Bendor and Wang, 2006).

### Sensory-motor interactions in speech perception, production, and imitation

At the neural level, the typical networks for speech production and perception were observed, in agreement with previous studies on vowel production and perception (Özdemir et al., 2006; Sörös et al., 2006; Terumitsu et al., 2006; Brown et al., 2008; Ghosh et al., 2008; Grabski et al., 2013). Our results also concord with previous studies on voluntary imitation of speech (Damasio and Damasio, 1980; Caramazza et al., 1981; Bartha and Benke, 2003; Molenberghs et al., 2009; Irwin et al., 2011; Reiterer et al., 2011) and fast overt repetition (Peschke et al., 2009), about the involvement of brain regions of the dorsal stream in deliberate imitation processes. Contrary to the “direct matching hypothesis,” however, we did not observe the whole dorsal stream, and the IFG in particular, to be overall significantly more activated during deliberate imitation of speech (Irwin et al., 2011). In our results, only four ROIs of the dorsal stream: the auditory cortex and Wernicke's area, bilaterally, were found to vary significantly in activation from the passive perception task to the perception step of deliberate imitation. Unexpectedly, greater activation was observed for the passive perception task. Nevertheless, several ROIs of the dorsal stream, including the right SMG, showed an activity that correlated negatively with the degree of imitation during the perception step of the imitative process. Peschke and colleagues (2009) also identified such a region in the right inferior parietal area, though with a positive correlation.

The first questions addressed in this study were to determine whether phonetic convergence relied on the same mechanism and neural network as deliberate imitation, and to what extent brain regions related to sensori-motor integration were involved in that potentially shared network. Neither the whole brain analysis nor the ROI analysis showed any significant modulation in brain activation between the two tasks of deliberate and unconscious imitations (see Tables 4, 5). Furthermore, the ROI analysis revealed that the activation of several regions in the dorsal stream—the auditory cortex and the SMG, bilaterally, as well as the left Wernicke area—negatively correlated with the degree of imitation, during the perception step of the imitation processes. All these observations support the idea that both deliberate and unconscious imitations are based on the same mechanism and neural network, involving regions of the dorsal stream. Unlike Leslie et al. (2004) who compared deliberate and unconscious face imitation, we did not observe a right lateralization in unconscious speech imitation, and more bilateral activations for deliberate speech imitation, which would support the idea of a “voice mirroring system” in the right hemisphere, as they suggested for face imitation.

Another question concerned the steps of the perception-production process at which the imitative process occurs: is imitation included in the perception process, in the production one, or in both? The ROI analysis revealed that the activation in several regions of the dorsal stream was significantly modulated between vowel production and perception reference tasks, and both the perception and production steps of unconscious imitation—in the auditory cortex and Wernicke's area, bilaterally, for the perception step; in the right auditory cortex, the supramaginal gyrus, and the left insula for the production step. In the case of deliberate imitation, however, significant changes in activation were also found in these ROIs, but only for the perception step, as compared to the vowel perception reference task. Finally, ROIs in the dorsal stream whose activation correlated with the degree of imitation were found for the perception step of imitative processes only. No such region was found for the production step, or for the whole imitative processes. These observations support the idea that (1) the imitation process requires both perception and production steps of the sensori-motor loop, and that (2) the degree of imitation is determined by processes occurring during the perception step. The fact that the degree of imitation is determined by processes occurring during the perception step supports the hypothesis that perception intrinsically includes an automatic update of sensori-motor representations from the speech inputs.

A last question dealt with the degree of control and consciousness that one can have on phonetic convergence and its inhibition. The behavioral results of this study showed that phonetic convergence can be inhibited to some extent. A great inter-speaker variability was observed: some speakers were able to inhibit this unconscious imitation completely (or even with an overcompensation), others only partially, while some speakers could not inhibit it at all. At the neural level, no additional region or network, out of the typical networks of speech production and perception, appeared to be specifically involved in imitation inhibition. It is worthwhile noting that a significant activation was observed during the perception step of deliberate and inhibited imitation in the dorsolateral prefrontal region BA46, an area commonly associated with attention, resource allocation, and verbal self-monitoring (Indefrey and Levelt, 2004). That region is also known to have connections with temporal areas and to play a role in auditory processes (Romanski et al., 1999). However, the activity of that region was not found to be significantly greater in deliberate and inhibited imitation, as compared to passive perception or unconscious imitation, which does not enable us to speculate further on its role in imitation processes. On the contrary, modulated activation was observed in the left insular cortex, a brain region involved, amongst other functions, in self-awareness and inter-personal experience. This is consistent with previous studies showing that resisting motor mimicry involves cortical areas that are required to distinguish between self-generated and externally triggered motor representations (Brass et al., 2003, 2005; Spengler et al., 2010).

## Conclusions and perspectives

The different behavioral and neural observations of this study support the hypothesis that phonetic convergence may not only be driven by social or communicative motivations, but that it may primarily be the consequence of an automatic process of sensorimotor recalibration. This has some important implications on speech production and perception, for the comprehension of how internal models and phonetic representations are learnt and updated. Indeed, many previous studies had shown how speakers modify their speech production to compensate for perturbations of their auditory or proprioceptive feedback (Abbs and Gracco, 1984; Houde and Jordan, 1998; Jones and Munhall, 2000; Villacorta et al., 2007; Shiller et al., 2009; Cai et al., 2010). After-effects of these compensations were observed on both speech production and perception (Nasir and Ostry, 2009; Shiller et al., 2009), reflecting an update of sensori-motor representations, in response to modifications of the sensory feedbacks. Complementory to these studies, our experiment brings new arguments supporting the idea that sensorimotor representations and internal models that map speech motor commands onto their sensory consequences are continuously updated, not only from the comparison between sensory feedbacks and the predicted consequences of actions, but also from the comparison between our own production and external speech inputs provided by others. This idea of “comparison” was explored by several neurofunctional studies that suggested the existence of an “auditory-error module,” supposed to be located in the posterior STG, more specifically in the planum temporale (Tourville et al., 2008). The same authors also proposed the existence of a “somatosensory-error module,” assumed to be located in the SMG and the left anterior Insula, modulated when the somatosensory feedback is perturbed (Golfinopoulos et al., 2011). Interestingly, these regions are exactly those whose activation was modulated between the different imitative tasks of our study, and whose activation correlated significantly with the degree of imitation.

This possible involvement of sensorimotor recalibration processes also has implications on the communicative and social aspects of phonetic convergence. Imitation may facilitate communication not only by improving our likeability or our intelligibility for the interlocutor, but also by helping *us* to better understand our interlocutor (his/her feelings, attitudes and speech). Thus, it was shown that imitating someone else's accent improves after our appreciation of this accent (Adank et al., 2013), or that covert imitation facilitates the prediction of upcoming words in sentences in adverse listening conditions (Adank et al., 2010) and to some more limited extent, the recognition of single words (Nguyen et al., 2012).

From these findings, the involvement of sensori-motor recalibration processes in phonetic convergence, and its potential explanation of higher-level communicative and social effects (inter-individual differences and phonetic talent, i.e., the ability to learn a second language, empathy and likability, intelligibility enhancement, …) remain to be investigated in future studies.

### Conflict of interest statement

The authors declare that the research was conducted in the absence of any commercial or financial relationships that could be construed as a potential conflict of interest.
